# Can Standardisation of the Public Assessment Report Improve Benefit-Risk Communication?

**DOI:** 10.3389/fphar.2020.00855

**Published:** 2020-06-17

**Authors:** Andrea Keyter, Sam Salek, Shabir Banoo, Stuart Walker

**Affiliations:** ^1^Department of Clinical and Pharmaceutical Sciences, School of Life and Medical Sciences, University of Hertfordshire, Hatfield, United Kingdom; ^2^South African Health Products Regulatory Authority, Johannesburg, South Africa; ^3^Right to Care and Faculty of Health Sciences, University of Witwatersrand, Johannesburg, South Africa; ^4^Centre for Innovation in Regulatory Science, London, United Kingdom

**Keywords:** benefit-risk assessment, regulatory decision-making, public assessment reports, ZAPAR, South Africa

## Abstract

**Background:**

National regulatory authorities (NRAs) make the decision to register a medicine based on an assessment of its benefits and risks and publicly available assessment reports are used as a tool to communicate the basis for the decision. The Universal Methodology for Benefit-Risk Assessment (UMBRA) has also been used to effectively communicate the basis of regulatory decisions. Many NRAs in emerging markets place reliance on the public assessment reports (PARs) of reference agencies to inform about their own regulatory decisions. However, PAR users often criticise the redacted nature of PARs and may be challenged in identifying key benefits and risks, value judgements, and benefit-risk (BR) trade-offs.

**Methods:**

PARs for ertugliflozin l-pyroglutamic acid, erenumab, and durvalumab published by regulatory bodies in Australia, Europe, Canada, and the United States were compared with the validated UMBRA Benefit-Risk Template to evaluate the BR decision documentation. Published validation of UMBRA included report of a consortium of four regulatory authorities in Australia, Canada, Switzerland, and Singapore indicating that their clinical assessment templates were modified to align with the UMBRA approach. A focus group discussed the use of PARs as potential knowledge management tools for stakeholder understanding of regulatory decision making. The South African Health Product Regulatory Authority (SAHPRA) approach to document and communicate the BR decisions was evaluated.

**Results:**

Results indicate key elements to include in the PARs including regulatory history, an effects table and a record of the strengths and uncertainties for each benefit and risk. Focus group participants agreed that a harmonised PAR template would support improved regulatory decision-making transparency. SAHPRA communication of BR decisions could be improved through the use of the UMBRA BR Template as a guidance for BR assessment and the basis of the South Africa public assessment report format.

**Conclusion:**

SAHPRA's use of a structured template that supports transparent and quality decision making could have a major impact in ensuring consistency in the BR assessment of new medicines. The implementation of this effective approach for communicating BR decisions will advance agency goals of being a trusted, responsive, accountable regulatory body in which all healthcare stakeholders may rely on with confidence.

## Introduction

National regulatory authorities (NRAs) are responsible for making the decision to register a medicine based on an assessment of its overall benefits and risks. Often the benefit-risk (BR) balance, which ideally includes an account of the uncertainties and risks and relevant stakeholder perspectives (McAuslane et al., 2017) is at the core of the regulatory decision to register a medicine (Pignatti et al., 2015). Regulators, academics, and the pharmaceutical industry have recognised the need for a common, structured, systematic approach to the BR assessment of medicines, which may be used during the review of an application for the registration of a medicine and for communicating the results of the review (Walker et al., 2011). A number of frameworks for BR assessment have been developed over the past several years (Walker et al., 2014). Many of these frameworks have incorporated mechanisms to support the systematic processing of data prior to making the regulatory decision (Walker et al., 2011) and featured structured, coherent, comprehensive approaches to BR assessment (Pignatti et al., 2015). While differences amongst these frameworks exist, the principles of “defining the decision, agreeing on the requisite properties of the treatments being considered, assessing the trade-offs among these properties and making defensible transparent decisions” were common (Levitan et al., 2014).

**Figure 1:**
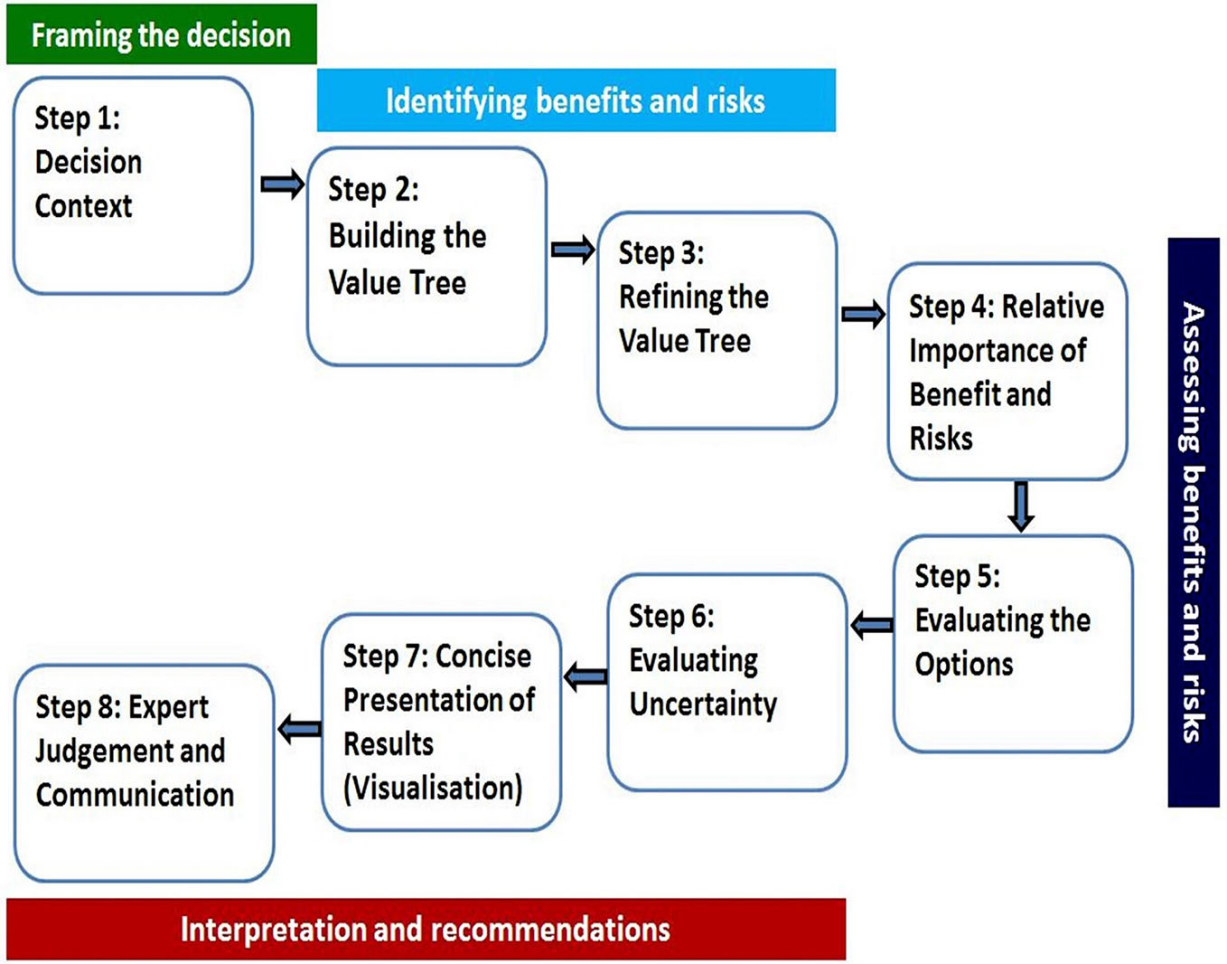
The UMBRA eight-step Benefit-Risk Framework.

A universal BR assessment framework that incorporated the existing frameworks was developed (Walker et al., 2014) and validated (McAuslane et al., 2017). The validation of the framework by McAuslane et al., 2017 further described that a consortium of four regulatory authorities, the Australian Therapeutic Goods Administration (TGA), Health Canada, Swissmedic, and Singapore Health Sciences Authority (HSA) requested support in the development of a benefit-risk framework and the template that was used by all four authorities and that would enable joint shared reviews to maximize resources. Notably, the agencies indicated that their clinical assessment templates were modified to align with the UMBRA 8-step framework approach (**Figure 1**). The Universal Methodology for Benefit-Risk Assessment (UMBRA) is an acceptable overarching BR framework (**Figure 1**) (Leong et al., 2015) that provides a template that may be used during the review and that documents the elements considered to be essential in the assessment of benefit and risk (Leong et al., 2014). The UMBRA BR Template is considered useful in collating the conclusions of the BR decisions (Leong et al., 2015) and could be used to effectively communicate the basis for the regulatory decision to register a medicine.

In an effort to ensure transparency and accountability, some NRAs publish their assessment reports to communicate the regulatory decision in a clear and understandable manner for consideration by the public. Public assessment reports (PARs) provide information about how the NRA has assessed the benefits and risks of a medicine (Raynor and Bryant, 2013). PARs usually include information pertaining to the data submitted to the NRA for evaluation as well as the conclusions made by the NRA (Raynor and Bryant, 2013). PARs are published in the public domain by NRAs to document the basis and justification for the regulatory decision and to promote transparency (Leong et al., 2014).

Results from a previous study (Leong et al., 2015) have demonstrated that making use of a BR framework enforced a structured, documented discussion and contributed to the improved quality of communication in terms of transparency and consistency (Leong et al., 2014).

Ensuring transparency in decision making and documenting regulatory decisions in a structured systematic manner promotes an enhanced understanding of the basis for a regulatory decision and the rationale for the inclusion or exclusion of benefits and risks and the determinants of the consequent BR balance (Leong et al., 2014). Many NRAs in emerging markets place reliance on the PARs of reference agencies to inform their own regulatory decisions (Ward, 2019). Users of PARs often criticise the redacted nature of the PARs and have experienced challenges in identifying the key benefits and risks that underlie the decisions made by reference agencies as well as the value judgements and the trade-offs between the benefits and risks (Raynor and Bryant, 2013). This study aims to review the PARs available in the public domain against the UMBRA BR Template using a case study approach. This is also the first review carried out to evaluate the approach initiated by South African Health Product Regulatory Authority (SAHPRA) to document and communicate the BR decision.

### Study Objectives

The main objectives of this study were to:

- use a case study approach to compare the publicly available assessment reports of ertugliflozin l-pyroglutamic acid, erenumab, and durvalumab recently published by the Australian Therapeutic Goods Administration (TGA), the European Medicines Agency (EMA), Health Canada, and the United States Food and Drug Administration (USFDA) against the validated UMBRA BR Template and to determine whether the BR decision has been documented in a systematic and structured manner;
- conduct a focus group discussion to explore the use of PARs as potential knowledge management tools for stakeholders in understanding a reference agency's decision making; and
- develop recommendations for SAHPRA for the implementation of an effective approach for communicating BR decisions.

## Methods

The authors' institutions do not require ethics approval for the type of study reported here.

### Case Study Comparing the Public Assessment Reports From Four Reference Agencies With the UMBRA BR Template

The PARs for three new active substances (NASs), including ertugliflozin l-pyroglutamic acid, erenumab, and durvalumab, recently published by four reference agencies were compared with the validated UMBRA BR Template (Walker et al., 2014). The four reference agencies and report formats selected for the study included the TGA Australian Public Assessment Report (AusPAR), EMA: European Public Assessment Report (EPAR), Health Canada Summary Basis of Decision (SBD), and the USFDA (Summary Review). These agencies were selected based on a long history of established regulatory processes, global recognition of regulatory standards, and the availability of their assessment reports in the public domain. The NASs (**Table 1**) were selected based on their relevantly recent and similar approval dates by the four reference agencies who are currently the only NRAs producing PARs.

**Table 1:** Public assessment reports of new active substances selected for comparison with the UMBRA Benefit-Risk template.

Active Pharmaceutical Ingredient	Indication	TGA Approval Date	EMA Approval Date	Health Canada Approval Date	USFDA Approval Date
Ertugliflozin l-pyroglutamic acid	Selective inhibitor of the sodium-dependent glucose cotransporters (SGLT) indicated for Type II Diabetes	14/05/2018	21/03/2018	09/05/2018	19/12/2017
Erenumab	Analgesic indicated for treatment of migraine	28/06/2018	26/07/2018	01/08/2018	17/05/2018
Durvalumab	Human immunoglobulin G1 kappa (IgG1κ) monoclonal antibody indicated for locally advanced or metastatic urothelial carcinoma	02/10/2018	21/09/2018	03/11/2017	01/05/2017

EMA, European Medicines Agency; NASs, new active substances; PARs, Public Assessment Reports; TGA, Australian Therapeutic Goods Administration; UMBRA, Universal Methodologies for Benefit-Risk Assessment; USFDA, United States Food and Drug Administration.

The PARs were retrieved online for each of the NASs. The comparison of the PARs for the three NASs prepared by the four reference agencies was conducted by comparing the information documented within the PARs against the various section headings of the UMBRA BR Template and tabulating the findings. This was carried out by the principal author and validated by the other members of the research team.

### Evaluation of the Approach Initiated by SAHPRA to Communicate the BR Decisions

The approach initiated by SAHPRA to document and communicate the BR decisions was evaluated. Since SAHPRA does not currently produce PARs, the following guidelines and templates used by SAHPRA to support the review of the quality, safety, and efficacy of NASs were compared against the section headings of the UMBRA BR Template: Guideline 2.09 Clinical Guideline (version 2, published in July 2019) (South African Health Products Regulatory Authority, 2019a); Guideline 6.31 Summary of Critical Regulatory Elements (SCoRE) Document (version 1, published in July 2019) (South African Health Products Regulatory Authority, 2019b) and the SCoRE template; the Clinical Full Review Report Template (CRT) (January 2019); and the SAHPRA Guideline for Clinical Reviewers (March 2019). This study was designed to be exploratory in nature and the results of the study provided qualitative interpretations related to the study objectives.

### Focus Group

A focus group was conducted in Tysons Corner, Virginia, United States in June 2019. The group comprised 15 participant representatives of regulatory authorities, the pharmaceutical industry, academia, and patient groups from different jurisdictions; a moderator responsible for facilitating the discussion and a rapporteur who was responsible for consolidating the results and reporting the outcomes. The remit of the focus group was to consider: “Public assessment reports—Are these good knowledge management tools for stakeholders such as other regulatory authorities, health technology assessment agencies, companies, and patients in understanding an agency's or company's decision making? If not, how can they be improved?” A brief guide was prepared for the focus group and this described the discussion topic, provided background information and a list of relevant questions and issues, and outlined the objectives for the discussion (**Appendix 1**).

## Results

For the purpose of clarity, the results are presented in three parts:

- Part I—Comparison of the four reference agency PARs against the validated UMBRA BR Template
- Part II—Review of the approach initiated by SAHPRA to document and communicate the BR decision
- Part III—Outcomes of the focus group

### Part I—Comparison of the Four Reference Agency PARs Against the Validated UMBRA BR Template

The TGA, EMA, Health Canada, and USFDA produce publicly available assessment reports to document the agency's decisions for product registration. The formats of these reports have been previously studied (Leong et al., 2014) and found to be generally similar and comparable to the format of the UMBRA BR Template (Walker et al., 2014). Three of the four agency PARs made provision for a documented benefit-risk assessment of the product. These included the TGA AusPAR (Section VII. Overall conclusion and risk/benefit assessment); the EMA EPAR (Section 3. Benefit-Risk Balance); and the USFDA (Summary Review: Section 1 Benefit Risk Assessment). The PARs produced by each of the four agencies followed a similar format and were comparable for each of the three products (durvalumab, erenumab, and ertugliflozin l-pyroglutamic acid) selected for the case study. The results of the three PARs produced by each of the four agencies were compared against the UMBRA BR Template as well as the current approach by SAHPRA in their regulatory review (**Table 2**).

**Table 2:** Comparison of TGA, EMA, Health Canada, and USFDA PARs and the SAHPRA BR appraisal with the UMBRA BR Template.

UMBRA BR Template: Content		TGA (AusPAR)		EMA (EPAR)		HC (SBD)		USFDA (Summary Review)		**SAHPRA appraisal of BR - SCORE**	
**1.1**	**Background (Decision context)**										
1.1.1	Specify proposed therapeutic indication		Section I. Introduction to product submission – Product background		Section 3.1.1 Disease or condition		Section 1 What was approved		Section 1: Benefit-risk integrated assessment		Not available
1.1.2	Treatment options evaluated		Section V. Clinical findings – Current treatment options		Section 3.1.2 Available therapies and unmet medical need		Section 2 Why was < product> approved?		Section 1: Benefit-Risk Dimensions – Current treatment options		CRT: Section 4.3.1
1.1.3	Unmet medical need		Section V. Clinical findings – Clinical Rationale		Section 3.1.2 Available therapies and unmet medical need		Not available		Section 1: Benefit Risk Dimensions – Analysis of conditions		Not available
1.1.4	Local clinical guideline or other issues		Not available		Section 3.1.2 Available therapies and unmet medical need		Not available		Not available		Not available
1.1.5	Previous review of active substance by the agency		Section I. Introduction to product submission – Regulatory status		Section 1.1 Submission of the dossier		Post-authorization Activity Table		Not available		CRT: Section 3
1.1.6	Reference agency regulatory history		Section I. Introduction to product submission – Regulatory status		Not available		Not available		Not available		CRT: Section 3 2.09: Section 4.2.6
**2.1**	**Overall summaries**										
2.1.1	Quality conclusion		Section III. Quality findings – Quality summary and conclusion and Section VII. Overall conclusion and risk/benefit assessment – Quality		Section 2.2.5 Conclusions on the chemical, pharmaceutical and biological aspects		Section 7.3: Quality Basis for Decision		Section 3: Product Quality		6.31: Section 2
2.1.2	Non-clinical conclusion		Section IV. Non-clinical summary and conclusion and Section VII. Overall conclusion and risk/benefit assessment – Nonclinical		Section 2.3.7 Conclusion on the non-clinical aspect		Section 7.2: Non-Clinical Basis for Decision		Section 4: Nonclinical Pharmacology/Toxicology		CRT: Section 4.2 6.31: Section 1.1
2.1.3	Human pharmacology conclusion		Section IV. Pharmacology and Section VII. Overall conclusion and risk/benefit assessment – Pharmacology		Section 2.4.5 Conclusions on clinical pharmacology		Section 7.1: Clinical Basis for Decision – Pharmacology		Section 5: Clinical Pharmacology		CRT: Section 4.1
2.1.4	Assessment of ethnic factors		Section V. Clinical findings – Evaluator's conclusions on safety/Special Populations		Section 2.6 Safety in special populations		Section 2: Why was < product> approved?		Not available		CRT: Section 4.3.1
**3.1**	**Clinical study summary**		Section V. Clinical findings – Contents of the clinical dossier		Section 2.4 Clinical Aspects Section 3.1.3 Main clinical studies		Section 7.1: Clinical Basis for Decision – Clinical Efficacy		Section 7: Clinical/statistical efficacy and Section 8: Safety		CRT: Section 4.3.1
**3.2**	**Clinical conclusion**		Section V. Clinical findings and Section VII. Overall conclusion and risk/benefit assessment – Clinical		Section 2.5.4 Conclusions on clinical efficacy and Section 2.5.6 Conclusions on clinical safety		Section 7.1: Clinical Basis for Decision		Section 7: Efficacy Conclusion and Section 8: Safety Conclusion		CRT: Section 4.3.2
**4.1**	**Risks: Overall summary**		Section V. Clinical findings: First and second round risk assessment		Section 2.6 Clinical Safety - Adverse events and Section 3.4 Unfavourable effects		Not available		Section 1: Benefit-Risk Dimensions – Risk and Section 8: Safety – safety conclusions		Not available
**5.1**	**Identified benefits and risks**										
5.1.1	Benefits documented: Listing of all benefits, and justification for inclusion and exclusion		Section V. Clinical findings: First and second round benefit assessment		Section 3.2 Favourable effects and Section 3.3 Uncertainties and limitations about favourable effects		Not available		Section 1: Benefit-Risk Dimensions – Benefit		Not available
5.1.2	Risks documented: Listing of all risks, and justification for inclusion and exclusion		Section V. Clinical findings:. First and second round risk assessment		Section 3.4 Unfavourable effects and Section 3.5 Uncertainties and limitations about unfavourable effects		Not available		Section 1: Benefit-Risk Dimensions – Risk and risk management		Not available
**6.1**	**Weighting and valuing of benefits and risks**		Not available		Section 3.7.1 Importance about favourable and unfavourable effects		Not available		Not available		Not available
**7.1**	**Conclusion**										
7.1.1	Effects table and conclusion: Listing the relative importance and valuing the options of the effects of each benefit and risk and commenting on any strengths or uncertainty		Not available		Section 3.6 Effects table		Not available		Not available		Not available
7.1.2	For negative benefit–risk balance, discussion on the harm		Section VII. Overall conclusion and risk/benefit assessment – Risk-benefit analysis		Section 3.7.2 Balance of benefits and risks		Not available		Section 1: Benefit-Risk Dimensions – Risk and risk management		Not available
7.1.3	Discussion on evolution of the benefit-risk balance		Section VII. Overall conclusion and risk/benefit assessment – Risk-benefit analysis		Section 3.7.1 Importance about favourable and unfavourable effects		Not available		Section 1: Benefit-risk integrated assessment		Not available
7.1.4	Evaluation of the pharmacovigilance plan and risk minimisation plan		Section VI. Pharmacovigilance findings and Section VII. Overall conclusion and risk/benefit assessment – RMP		Section 2.6 Risk management plan and Section 2.7 Pharmacovigilance		Section 2: Why was < product> approved? And Section 5: What post-authorization activity has taken place for < product>?		Section 1: Benefit-Risk Dimensions - Risk and risk management and Section 12/13/14: Postmarketing recommendations		CRT: Section 4.4 6.31: Section 1.1
7.1.5	Discussion on outstanding issues and other significant information (hearings, advisories, patients, consumers, stakeholder inputs)		Section VII. Overall conclusion and risk/benefit assessment – Specific conditions of registration applying to these goods and Summary of issues		Section 3.7.1 and Section 4 Recommendations		Section 4: What follow-up measures will the company take?		Section 12/13/14: Postmarketing recommendations		Not available
7.1.6	Discussion on need for further studies		Section VII. Overall conclusion and risk/benefit assessment – Specific conditions of registration applying to these goods and Summary of issues		Section 3.7.3 Additional considerations on the benefit-risk balance		Section 4: What follow-up measures will the company take?		Section 12/13/14: Postmarketing recommendations		Not available
7.1.7	Any other information relevant to the benefit-risk decision		Section VII. Overall conclusion and risk/benefit assessment – Risk-benefit analysis		Section 3.7.3 Additional considerations on the benefit-risk balance		Section 3: What steps led to the approval of < product>? (Limited) (Reference made to reference agency PARs from USFDA and EMA)		Section 1: Benefit-risk integrated assessment		Not available
7.1.8	Conclusion on the benefit-risk balance for proposed indication		Section VII. Overall conclusion and risk/benefit assessment – Concluding remarks		Section 4 Recommendations		Section 2: Why was < product> approved?		Section 1: Benefit-risk integrated assessment		CRT: Section 4.4 6.31: Section 1.1
7.1.9	Recommendation indication		Section VII. Overall conclusion and risk/benefit assessment – Outcome		Section 4 Recommendations		Section 7.1: Clinical Basis for Decision – Indication		Section 1: Benefit-risk integrated assessment		Not available
7.1.10	Indicate if the approved indication is the same as the reference agencies used for this review		Not available		Not available		Not available		Not available		Not available

Legend				Available			Available but information is limited				Not available

AusPAR, Australian Public Assessment Report; BR, benefit-risk; CRT, Clinical Report Template; EMA, European Medicines Agency; EPAR, European Public Assessment Report; PARs, Public Assessment Reports; SBD, Summary Basis of Decision; TGA, Therapeutic Goods Administration of Australia; UMBRA, Universal Methodology for Benefit-Risk Assessment; USFDA, United States Food and Drug Administration.

#### TGA AusPAR

The AusPAR for durvalumab was not available at the time of the study and the results reflected in **Table 2** were based on the comparison of the AusPARs produced for erenumab and ertugliflozin l-pyroglutamic acid against the UMBRA BR Template. The assessment of ethnic factors was not well documented within the AusPAR. The list of phase I, pivotal, supportive, and ongoing studies was provided but a record of the key benefits or risks identified in the studies was not included. A narrative describing the risks of the product was available however, the summary of risks was not easily identified and a table of the pooled overall incidence of events was not provided. Section V of the AusPAR provided a documented clinical rationale for the use of the product but did not provide documented justification for the decision as to whether the product fulfilled an unmet medical need. The assessment of the benefits and the risks was documented in Section V (clinical findings). The reviewed benefits and risks selected for inclusion in the assessment were not explicitly listed, were not assessed in terms of relative importance, and were not valued. The justification for the inclusion or exclusion of the benefits and risks was not documented. The reviewer's considerations in terms of the benefit-risk assessment were provided as a narrative discussion in Section VII, however a clear conclusion on the benefit-risk being positive or not for the proposed indication was not provided.

#### EMA EPAR

The regulatory history of the product with regard to its assessment by a reference agency was not documented. The list of clinical trials conducted was provided but a record of the key benefits or risks identified in the studies was not included. The EPAR documented the favourable and unfavourable effects of the product as well as the associated uncertainties and limitations of these effects; however, it did not provide a record of the benefits and risks that were reviewed and the reasons for their inclusion or exclusion in the benefit-risk assessment of the product. An effects table was provided in Section 3.6 of the EPAR and the importance of favourable and unfavourable effects was discussed in Section 3.7.1. The assignment of weighting (relative importance) of each of the benefits and risks identified and the valuing of the options of the effects was not explicitly recorded. The EPAR did not provide a record of the expected evolution of the benefit-risk balance over time.

#### Health Canada Summary Basis of Decision (SBD)

The SBD did not make provision for the explicit assessment and documentation of the benefit-risk balance. Ethnic considerations were not routinely documented. The clinical study summary and associated benefits and risks identified in each study were not documented. Also, the overall summary of risks, the benefits and risks, and the effects table were not available. The relative importance and values of benefits and risks were not documented; justification for their inclusion or exclusion was not recorded and no comments were made regarding the strengths and uncertainties of the benefits and risks that were included in the review. No information was available to describe the expected evolution of the benefit-risk balance over time. The SBD provided limited information to describe the outstanding issues and how these issues were to be addressed. For example, the requirements for additional follow-up measures or specific obligations, the need for further product development, as well as further studies to improve the benefit-risk balance were not documented.

#### US FDA Summary Review

While the summary review did not document the justification for the decision as to whether the product fulfilled an unmet medical need, an analysis of the condition was provided and included related evidence and uncertainties as well as brief conclusions and reasons justifying the need for the treatment of the condition. The summary review did not specify any local clinical guideline or other issues which needed to be considered to contextualise the decision. The regulatory history of the product with regard to a previous assessment by the agency or by another reference agency was not documented. The consideration of ethnic factors was not recorded. The clinical/statistical efficacy and safety were documented in Section 7 and Section 8, respectively. A clinical study summary providing a highlight of the study designs, treatments and the conclusions, identifying the key benefits or risks, was not included. In line with the findings noted by Leong et al. (2014), the summary review had not been amended to make provision for a record indicating which benefits and risks were reviewed by the agency or the rationale as to which were subsequently included or excluded. The summary review did not include a record of the relative importance assigned to each benefit and risk and did not make provision for valuing the options and commenting on the strengths and uncertainties for each benefit and risk identified. The benefit-risk integrated assessment was available but did not necessarily describe how the benefit-risk balance was expected to evolve over time for example in the event that late side effects emerged or if long-term efficacy decreased.

### Part II—Review of the Appraisal Initiated by SAHPRA to Document and Communicate BR Decisions

The appraisal initiated by SAHPRA to document and communicate the BR decisions to sponsors was evaluated by comparing the SAHPRA guidelines and templates, used to support the assessment of NASs, against the section headings of the UMBRA BR Template (**Table 2**).

A description of the treatment options evaluated (Section 1.1.2 of the BR Template) was included in Section 4.3.1 of the clinical unit full report template (CRT) but was limited to comments on the stratification between treatment-naïve and treatment-experienced patients and/or stratification between patients previously exposed to different treatment options and how it related to the intended use of the medicine as described in the professional insert. Information pertaining to the review of the active substance by a reference agency (Section 1.1.6 of the BR Template) was included in Section 3 of the CRT, however the information requested was limited to an indication of the registration status of the medicine with regulators with which SAHPRA aligns itself. An assessment of ethnic factors (Section 2.1.4 of the BR Template) was included in Section 4.3.1 of the CRT but was limited to comments on patient demographics stratified by ethnic groups and how this was related to or affected the intended use described in the professional insert. The CRT: Section 4.4 made provision for a summary of the BR analysis and assessors were required to provide information pertaining to the risk management plan or risk minimisation measures and implementation plan. The clinical study summary was required to be presented as a narrative within the CRT and was limited in that the key benefits and risks identified in each clinical study were not documented. The benefits and risks were not listed, no effects table was available and again, the relative importance, valuing, and justification for inclusion/exclusion were not documented. The discussions on the harms, the evolution of the benefit-risk balance, outstanding issues, the need for further studies, the conclusion on the benefit-risk balance, and the recommended indication were not documented. An evaluation of the risk minimisation plan was only applicable for applications for abridged reviews and an evaluation of the pharmacovigilance plan was not documented.

The Clinical Guideline – 2.09 (South African Health Products Regulatory Authority, 2019a) confirmed that the applicant was required to provide the reference agency regulatory history to SAHPRA, however, this requirement was limited to applications for abridged reviews only. The internal SAHPRA Guidance for Clinical Reviewers (March 2019) provided instruction to SAHPRA reviewers on the required format and content of a full clinical review report. Clinical reviewers were required to ensure that review reports were sufficiently detailed to allow for secondary assessment by other expert clinical reviewers. During the review of clinical data, reviewers were required to comment as to:

- whether the BR balance at maximum dose was acceptable;
- the BR balance presented by the applicant;
- whether or not the suggested risk management plan and risk mitigation measures addressed the safety issues identified within the BR analysis of the safety information of the clinical studies;
- whether quality-of-life issues were addressed in the clinical studies; and
- the safety issues reflected in the periodic safety update report (PSUR) or periodic benefit-risk evaluation report (PBRER) or changes in the benefit-risk balance, risk management plan, and risk minimisation measures when a phase IV post-marketing study is submitted for a medicine that is registered by an NRA with which SAHPRA aligns itself.

While these requirements were listed in the internal SAHPRA Guidance for Clinical Reviewers (March 2019) as elements to be reviewed, provision was not made to document the reviewer's assessment of these elements within the CRT.

### Part III—Outcome of Focus Group Discussion

The focus group that was brought together included participants from the regulatory authorities, pharmaceutical industry, and academia. The outcome of the focus group that was held in Virginia in June 2019 resulted in recommendations for consideration in the use of PARs as potential knowledge management tools for stakeholders such as other NRAs, health technology assessment agencies, industry, society, and patients in understanding reference agency decision making. The participants identified the need for reference agencies producing PARs to ensure that regulatory decisions were documented in a structured and systematic manner. They agreed that a standardised PAR template would support improved transparency in regulatory decision making by aiding the understanding of how the regulatory decision was made and by allowing for easy comparison of the regulatory decisions made by different reference agencies. Participants further agreed that such an initiative would support the effective communication of regulatory decisions to NRAs that place reliance on the decisions made by these reference agencies. It was recommended that reference agencies should consider publishing PARs or releasing information related to negative regulatory decisions; that is, the rejection of an application for product registration, and for regulatory decisions made pertaining to applications for extension of product indications. The focus group concluded that the strengths of this work is that it compared the PARs produced by reference agencies against a structured, systematic BR template.

## Discussion

National regulatory authorities publish public assessment reports in an effort to enhance transparency and accountability in the regulatory decision-making process. In the public healthcare sector, the publication of PARs contributes towards building public confidence in the regulator and demonstrating the regulator's ability to ensure that available medicines are safe, effective, and of good quality. Patients may refer to PARs to better understand the benefits and harms associated with the medicines that have been prescribed to them and practitioners may use them to guide their decisions in selecting one treatment option over another (Leong et al., 2014). The pharmaceutical industry and applicants submitting dossiers to NRAs for medicine registration use such reports to better understand the basis of the regulatory decision and the regulator's rationale for supporting the final BR balance (Leong et al., 2014). Their availability allows stakeholders to better understand any differences in data interpretation and the regulatory opinions that may exist amongst NRAs in different jurisdictions (Leong et al., 2014). Other smaller NRAs, particularly in the emerging markets place reliance on reference NRAs or recognise the decisions of reference NRAs when making local decisions on BR and the local summary basis of the decision to register a medicine in their jurisdiction (McAuslane et al., 2017).

Pubic assessment reports have been recognised by various stakeholders as good knowledge management tools in understanding regulatory decision making. National regulatory authorities may have legislated duties to make certain information available in the public domain through the publication of PARs or may publish these to support the goals of enhanced public transparency (McAuslane et al., 2017). The preparation and publication of PARs may inherently contribute to the effective and timely documenting of regulatory decisions by NRAs to support regulatory performance efforts to build quality into regulatory decision making and maintain the consistency of decisions and scientific advice (Skerritt, 2019). Documenting the regulatory decision-making process including both internal and external decisions and commitments is crucial and may serve as a platform whereby past decisions may be used to inform future decisions in a consistent manner while contributing to evolved regulatory pathways that enlist accelerated review processes.

Currently, PARs, as they stand, cannot replace a review of the full dossier for those products previously reviewed by another competent authority. Therefore, a regulatory authority such as SAHPRA would need to have access to “assessment reports” if they were to adopt full reliance strategy. However, if a standardised PAR exists, they could use that as the basis of their review, which would mean that they would not have to carry out review of the full dossier. This approach in turn would have the benefits of reducing the review time, avoiding backlog, and reduce the increasing demand on resources.

Regulatory decision making unfolds through the assessment of benefits and risks and culminates in the final regulatory judgement on the BR balance. It is recognised that several structured approaches to performing the BR assessment exist (Leong et al., 2014; Levitan et al., 2014) through the identification of the initial set of clinical endpoints for the medicine under review and may be illustrated through the use of visualisation tools such as the value tree (Levitan et al., 2014). The importance of incorporating the perspectives of different stakeholders, notably that of the patient, has been emphasised as a result of the influence of patient-reported outcomes on the relevance of each endpoint for the decision and the consequent reassessment of the clinical endpoints within the value tree (Leong et al., 2014; Levitan et al., 2014; McAuslane et al., 2017). The data for such endpoints should be assessed and the relative importance should be assigned to each endpoint. This should be indicative of the relative clinical importance of the endpoint in order to support and contextualise the final decision in terms of the BR balance. Furthermore, the preparation of an effects table, listing the key benefits and harms has been demonstrated to support structured discussion through focused gap analysis and the identification of critical issues (Levitan et al., 2014). The decision-making process should also document the framing of the benefits and harms that should be assessed and the justification for their inclusion or exclusion should be recorded (Leong et al., 2014).

In the study conducted by Leong et al., 2014 it was noted that there were discrepancies in the information provided through the PARs prepared by reference NRAs when compared with the UMBRA BR Template. Since then, these NRAs have taken steps to enhance their PARs; however, the results of this case study indicate that these may be further improved to enhance communication of the BR decision to interested stakeholders. Currently, PARs do not contain the essential elements (i.e. redacted PARs) that should be included in order to identify the decision-making process. Therefore, as a result of this study it has been noted that the following key elements should be considered for inclusion in the PARs in order to effectively communicate the summary basis of the regulatory decisions and the key discussion points that lead to the BR decision to accept or reject the application for the registration of a medicine:

- A clinical study summary of the key benefits and risks identified in the clinical studies
- An effects table, listing each of the benefits and risks identified and a record of the justification for the inclusion of the benefits and risks assessed
- Documented assigned weighting (relative importance) of each of the benefits and risks, taking into consideration relevant stakeholder perspectives
- Documented valuing of the options and a record of the strengths and uncertainties identified for each benefit and risk
- A record of the expected evolution of the BR balance over time
- A record of the regulatory history of the product
- A record of the indication of the medicine in comparison with that approved by the reference agency

The study conducted by Leong and associates and the results of this case study confirm that the PARs prepared by the NRAs were similar in purpose, format, and context and supported the use of a universal template for documenting and communicating BR decisions (Leong et al., 2014). The UMBRA framework made provision for the listing of benefits and harms, assigning relative importance and valuing the options. It also provided a platform for structured discussion and a documented appraisal of the BR parameters through the use of a common language and presentation. Using the UMBRA BR Template would provide healthcare stakeholders with the clear understanding of the key messages presented by the NRA as the summary basis of the regulatory decision, using a format suitable for public consideration (Leong et al., 2014; Walker et al., 2014; McAuslane et al., 2017).

The UMBRA BR Template provides a mechanism for NRAs to document their BR assessment and build quality into their decision-making practices in a structured way as part of their efforts to ensure good review practices (World Health Organization, 2015; McAuslane et al., 2017). This approach could be used as an assessment template for NRAs wanting to enhance their BR assessment and could potentially serve as a guidance on BR assessment and a training tool for both regulatory reviewers and industry stakeholders responsible for the assessment of new medicines (McAuslane et al., 2017). Making use of this template as an outline for a PAR would enhance consistency in regulatory decision making and provide an effective tool for the review of past regulatory decisions. The UMBRA BR Template supports the clear articulation of each benefit and harm and contributes towards the ease of comparison of regulatory outcomes for medicines of the same class and the decisions by different NRAs for the same product (Leong et al., 2014; McAuslane et al., 2017).

The South African regulatory authority, SAHPRA, initiated an appraisal to ensure that the BR balance was considered during the review of NASs. This study has identified a number of deficiencies in the appraisal that has been initiated by SAHPRA. The current guidelines and report templates used by SAHPRA do not contribute fully to the comprehensive, structured, consistent evaluation of each of the benefits and harms and do not provide documented justification for the final decision on the BR balance or the decision to accept or reject the registration of the medicine.

National regulatory authorities worldwide, irrespective of size and expertise have or are considering the implementation of facilitated regulatory pathways; entering into work sharing arrangements with other NRAs and placing reliance on or recognising the regulatory decisions of other NRAs (Liberti, 2017; Liberti et al., 2018; Azatyan, 2019; Ward, 2019). In the light of the unavailability of a standardised PARs, which incorporate the relevant information to understand the decision-making process, then it would be of value for the agencies to have in place a “Memorandum of Understanding” in order to facilitate the availability of the assessment report.

A study by McAuslane and colleagues demonstrated that making use of a common approach to BR assessment and decision making was pivotal in the implementation of work-sharing models and in enabling the effective utilisation of information and expertise (McAuslane et al., 2017). Considering the drive by SAHPRA to embrace reliance models and their involvement in work sharing initiatives such as Zazibona, it may be valuable for the agency in South Africa to consider using a universal template and common approach to BR decision-making.

Key recommendations for SAHPRA for the implementation of an effective approach for communicating BR decisions should include:

1. Ensuring that the BR assessment is performed in a structured, systematic documented manner in alignment with good review practices in order to build quality into decision-making
2. Preparation and publication of a South African public assessment report (ZAPAR) in order to effectively communicate the BR decision to stakeholders and to ensure consistency, transparency, and accountability in regulatory decision making
3. Consideration of the UMBRA BR Template as guidance for BR assessment and as an outline for the ZAPAR which may further contribute toward:
  - Ease of comparison of regulatory decisions made by SAHPRA and other NRAs for the same medicine or for decisions made by SAHPRA for medicines in the same class
  - The review of past regulatory decisions to ensure consistency and objectivity in post-market assessments and product life cycle management
  - The use of documented BR assessments as a reference to facilitate expedited review times; as a result of better understanding of past decisions that may support faster decision making in line with goals of accelerated review times for NASs.

The implementation of an effective approach for communicating BR decisions by SAHPRA based on these recommendations should have a major impact on ensuring consistency in the BR assessment of NASs through the use of a structured template that supports transparent quality decision-making. Communicating the regulatory decisions of SAHPRA in the public domain will also enhance their goals of being a trusted, responsive, accountable regulator on which all stakeholders such as the industry and public may rely.

## Data Availability Statement

The datasets generated for this study are available on request to the corresponding author.

## Ethics Statement

Ethical approval was not required according to institutional guidelines/national legislation.

## Author Contributions

AK: Designed the study, analysed data, wrote manuscript. SS: Analysed the data, wrote the manuscript. SB: Analysed the data, wrote the manuscript. SW: Analysed the data, wrote the manuscript.

## Funding

AK, SS or SB did not receive any funding for the design, collection, analysis, or interpretation of data nor for the writing of the manuscript. SW was partly funded through unrestricted grant from Bill & Malinda Gates Foundation.

## Conflict of Interest

The authors declare that the research was conducted in the absence of any commercial or financial relationships that could be construed as a potential conflict of interest.

## Appendix 1 – Focus Group Guidance

**Table:**

Public assessment reports – Are these good knowledge management tools for other stakeholders such as other regulatory authorities, HTA agencies, companies and patients in understanding an agency's or company's decision making? If not, can they improve?

### Background to Decision Making

A public assessment report (PAR) should reflect the scientific conclusions reached by a regulatory agency at the end of the **evaluation** process and provide a summary of the grounds for granting a marketing authorisation for a specific medicine for human use. The utilisation of the information held within PARs are becoming important for different stakeholders, and PARs have a role in communicating medicines information beyond just the jurisdiction in which the decision is made. PARs are utilised for different purposes by different stakeholder:

- Companies—as a useful information source to identify and understand the evidence considerations regarding similar therapy areas; if a company or SME is moving into a new therapy area, PAR can provide insight into the development of similar medicines.
- HTA—in Europe the HTA and regulators have worked together to reduce inefficiencies by reusing the regulatory PAR in regard to Rapid Effectiveness Assessments.
- Other Regulators—As many regulators around the world move to reliance-based decision making where they look to leverage their process by utilising a reference agency's decision, this may be done through the use of redacted or unredacted assessment reports or through PARs.
- Patients—to gain an understanding of the medicine that has been approved regarding benefits and harms

PARs differ in the depth of information provided by an agency and not all agencies provide reports. However, these provide transparency and insight into aspects such as what evidence was considered regarding the benefit-harms-uncertainty balance, providing judgment on the methodological quality of the studies, and the relevance and significance of results.

Work undertaken in the past to look at the usefulness of PARs have concluded that although FDA reports are difficult to navigate they provide good information on both safety and efficacy whist the EPARS are easier to search and good for efficacy. Indeed, prior to the formalisation of the benefit risk frameworks by agencies a CIRS study was undertaken in 2012 and concluded that: compared with the elements that make up the BR template, existing PARs were inadequate regarding the listing of benefits and risks, the assigning of relative importance and values, visualisation and the utilisation of a detailed, systematic, standardised structure. The question is, have things changed since the adoption of agencies to standardise their individual approaches for articulating the benefits and harms of a medicine? Therefore, in terms of publication of information on medicines, this should be driven towards accessibility and usability by stakeholders, the question for this syndicate is therefore, “are PAR good knowledge management tools in understanding an agency's decision making?” If not, what would be of value to document?

Syndicate discussion:

The objectives are to:

1. Discuss public assessment reports and their potential role as knowledge management tools for other stakeholders in understanding an agency's or company's decision making?
2. Identify if public assessment reports contain enough information to understand the rationale for a decision, and if not, to suggest what would be of value to document; conversely, can a PAR be too detailed and complicated for a clear assessment?
3. *Make two or three recommendations as to the way forward for this topic*.

The Rapporteur's feedback report should include an outline of the main discussion points, the issues which have been discussed, and the recommendations. In structuring your recommendations, please consider recommendations that could be carried out in the **short term** and those which may be for more **long-term** consideration either directly by organisations or future research. Consider also one aspect of this topic that you could recommend CIRS pursue as a **research project**.

Please also consider identifying who you believe should be the actors for the recommendations; for example, CIRS, pharmaceutical companies, regulatory agencies, Centres of Excellence.

### Discussion Prompts

**PAR: How well do the PARs from the sources below fare regarding documentation of the following elements of a decision** (please rate as 1- Fit for purpose; 2-could be improved)

**Table:**

Key elements are systematically documented that:	FDA	EMA	TGA	Health Canada	Other agency, please specify
Provide both companies and other agencies clear understanding of the rationale of the decisions					
Define the **issues precisely, thereby reducing the ambiguity**					
Provide clarity where divergences may occur between agencies					
Key Elements about the decision that are included in the PAR					
providing the decision context					
options considered					
evidence and uncertainties					
Benefits, Risk/Harms, Uncertainties/trade offs					
Other—please specify					

- For any element rated a 2 – in what way could the information be improved?

### Maximizing the Use of PAR for Knowledge Management

- Are PARs of value for use within and across agencies and companies to help inform decisions?

please also consider this from the other stakeholder's perspective, patients, HTA, other regulatory agencies
  - If not, what are the issues (pros and cons) and how could these be addressed to maximize the use of PAR for knowledge management and decision support?

Please discuss, identify and recommend which information should be documented in PAR in a way that would better support stakeholder's knowledge management
